# Effect of Machine Settings on Ultrasound Assessment of B‐lines

**DOI:** 10.1002/jum.15581

**Published:** 2020-12-02

**Authors:** Isaac Matthias, Nova L. Panebianco, Mitchell G. Maltenfort, Anthony J. Dean, Cameron Baston

**Affiliations:** ^1^ The Department of Internal Medicine Section of Hospital Medicine; ^2^ The Department of Emergency Medicine; ^3^ The Department of Internal Medicine, Division of Pulmonary Allergy, and Critical Care, Hospital of the University of Pennsylvania Philadelphia Pennsylvania; ^4^ The Department of Biomedical and Health Informatics at the Children's Hospital of Pennsylvania Philadelphia Pennsylvania

**Keywords:** B‐lines, focal zone, gain, harmonics, lung ultrasound, settings

## Abstract

**Objectives:**

B‐lines are a lung ultrasound (LUS) artifact that often indicate pathology. Little is known about the optimal ultrasound machine settings to assess B‐lines. We compared settings typically used to evaluate B‐lines at our institution with adjusted settings based on recent studies.

**Methods:**

In order to determine typical settings for B‐line assessment, we retrospectively reviewed LUS images obtained at our institution. We then prospectively performed LUS with both typical and adjusted settings, using curvilinear and phased array probes, in 20 patients presenting to the emergency department with shortness of breath. The prospectively obtained clips were rated for quality and quantity of B‐lines by 14 clinicians with experience in LUS, with 1 assigned for typical settings “much greater,” 2 for typical settings “slightly greater,” 3 for both settings “similar,” 4 for adjusted settings “slightly greater,” and 5 for adjusted settings “much greater.”

**Results:**

Mean ratings and 95% confidence intervals significantly exceeded the null value of 3 for both B line quality (curvilinear probe: 4.68, 4.50–4.85; phased array probe: 4.02, 3.70–4.35) and B line quantity (curvilinear probe: 4.16, 3.84–4.49; phased array probe: 3.68, 3.41–3.96).

**Conclusions:**

B‐line quality and quantity were rated higher using adjusted settings based on recently published evidence than when using settings that are typically employed in our institution. Our findings suggest that B‐line assessment should be performed with focal zone at the level of the pleura, harmonics off, and gain increased in the far field.

**Abbreviations**
FAST: focused assessment with sonography for trauma
LUS: lung ultrasound
POCUS: point of care ultrasound
TGC: time gain compensation

## Introduction

B‐lines are a lung ultrasound (LUS) artifact consisting of vertical lines originating at the pleura and extending to the far field of the image.^1^ B‐lines are characteristic of common lung pathologies including cardiogenic pulmonary edema, interstitial lung disease, acute respiratory distress syndrome, pulmonary fibrosis, and others.^2^, ^3^, ^4^, ^5^, ^6^, ^7^, ^8^, ^9^ The test is noninvasive, rapid, repeatable, inexpensive, avoids ionizing radiation, and is easily learned.^10^, ^11^ B‐line assessment has become a core skill for point‐of‐care ultrasound (POCUS) in Emergency Medicine, Critical Care, Family Medicine, Internal Medicine, and many subspecialties.^12^, ^13^, ^14^, ^15^ Despite this, the dependence of B‐line images on technical aspects of image acquisition is not well understood.

Quantification of B‐lines is clinically important because it provides a tool to evaluate the severity of pulmonary edema and a dynamic measure of progression of disease. However, B‐line quantification is subject to variability in sonologist expertise, the time spent in evaluation, transducer type, the depth setting of the ultrasound screen, patient habitus, and underlying pulmonary conditions such as fibrosis.^16^ Potential operator factors relating to the LUS exam include probe orientation on the chest, angle of insonation, technique of B‐line counting, and number of intercostal spaces evaluated. Some of these have been systematically investigated.^16^, ^17^, ^18^, ^19^, ^20^, ^21^, ^22^

Beyond operator factors, little is known about the optimal ultrasound machine settings to view B‐lines. The 2012 LUS international consensus guidelines make no mention of settings for B‐line assessment, and studies that use B‐lines as a metric typically do not provide information on preset, focal zone, harmonics, or time gain compensation.^6^, ^15^, ^18^, ^23^, ^24^ Published studies have used a variety of presets, including cardiac, abdominal, and lung presets as set by the manufacturer.^8^ In Schmickl's recent in vitro study it is suggested that the optimal settings to view B‐lines include focal zone at the level of the pleura, harmonics off, and increased gain in the far field.^19^ While Schmickl found that his in vitro findings correlated with those of a single in vivo exam, it is uncertain whether this could be generalizable to the range of body types and B‐line findings encountered in clinical practice. In this paper we investigate how ultrasound machine settings affect sonologists' perception of B‐lines in a cohort of patients presenting to the ED with shortness of breath.

We have noted that many operators assess for B‐lines using default machine settings without adjustment, frequently using a phased array probe in an integrated cardiopulmonary exam. Accordingly, we used settings commonly used at our institution for comparison against published recommended settings. Specifically we compare images generated using “adjusted” settings based on published studies versus those “typical” settings.^19^, ^25^ The null hypothesis was that experienced sonologists would perceive no difference between B‐line images obtained using the two types of settings.

## Materials and Methods

### Study design

This study was performed in two phases at an urban academic medical center with an annual emergency department census of 72,000. First, to determine the typical practice at our institution we performed a retrospective review of emergency department POCUS B‐line images that had been obtained over a 6‐month period. The findings of this phase were used to determine the “Typical” settings to be used as a basis for comparison with the “Adjusted” settings based on published recommendations as discussed above. In the second phase, videos obtained from patients presenting to the emergency department with shortness of breath were prospectively recorded using both Typical and Adjusted settings and compared by experienced sonologists. The study was approved by the Institutional Review Board, which waived the requirement for written informed consent. All patients undergoing prospective ultrasound provided verbal informed consent.

### Phase 1: Retrospective assessment of ultrasound machine settings in clinical practice

A retrospective review was made of point‐of‐care cardiopulmonary ultrasound performed from January 2019 to June 2019 in the emergency department in our institution. When LUS clips evaluating B‐lines were present, we recorded the probe used and settings for preset, focal zone, frequency, and harmonics. We were unable to evaluate the time gain compensation (TGC) settings used, as these are not recorded in our image review software (QPath, Telexy, Maple Ridge, British Columbia). All ultrasound examinations in this study (in both the retrospective and prospective parts) were performed with a model M9 machine by Mindray North America, using a curvilinear probe with frequency range 1–5 megahertz (model C5‐1s) or a phased array probe with frequency range 1–5 megahertz (model SP5‐1 s).

**Figure 1:**
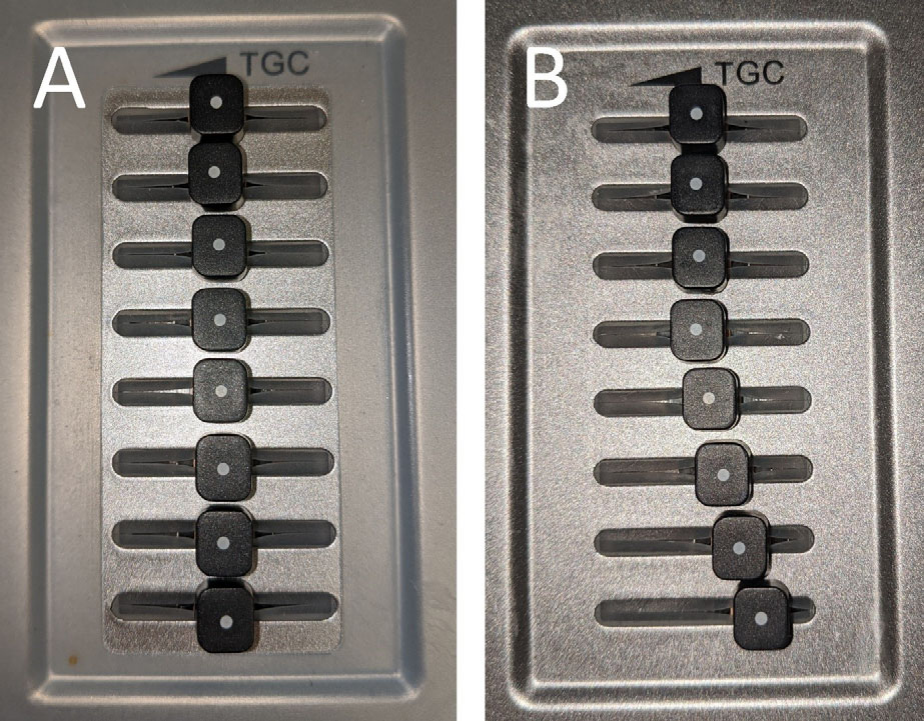
Time gain compensation adjustment. **A**, For the Typical setting, gain is set to the midpoint all levels of the image. **B**, In the Adjusted setting, gain is increased linearly in the far field half of the image.

As described further in the Results section, mean focal zone was 6.1 centimeters deep to the pleura, and harmonics were on in 86.8% of exams. We thus created a Typical setting to be used in the second, prospective phase of the investigation that consisted of focal zone 6 centimeters deep to the pleura, harmonics on, and, based on our experience that most sonologists do not adjust TGC for LUS, TGC at the midpoint at all levels of the image (Figure 1A). “Adjusted” settings, based on recent in vitro studies, were focal zone at the level of the pleura, harmonics off, and TGC increased linearly in the far field (Figure 1B). Other machine adjustments for both settings were otherwise identical, including lung preset, screen depth 16 centimeters, overall gain 50%, frequency at the mid‐range setting, dynamic range 130 decibels, spatial and temporal compound imaging off, and speckle reduction off.

### Phase 2: Prospective collection and rating of B‐line images

From September 2019 to November 2019 one investigator (I.M.) prospectively enrolled a convenience sample of patients presenting to the emergency department with a complaint of shortness of breath who were undergoing lung ultrasound. Patients with an intercostal space in the right upper chest that demonstrated three or more B‐lines using Adjusted settings were eligible.^1^ A curvilinear probe was placed in the region of the second intercostal space in the right midclavicular line, with probe marker oriented cephalad. To obtain an angle of insonation perpendicular to the pleura, the probe was fanned to produce the brightest and crispest appearing pleural line. A still image was taken, and the distance from the probe to the pleura was measured. After obtaining this image, two 10 second clips were recorded without moving the transducer, one with Adjusted, and the other with Typical settings. Immediately after these videos were obtained the same protocol was followed using a phased array probe in the same location and intercostal space. The etiology of patients' shortness of breath was made by chart review after discharge.

**Figure 2:**
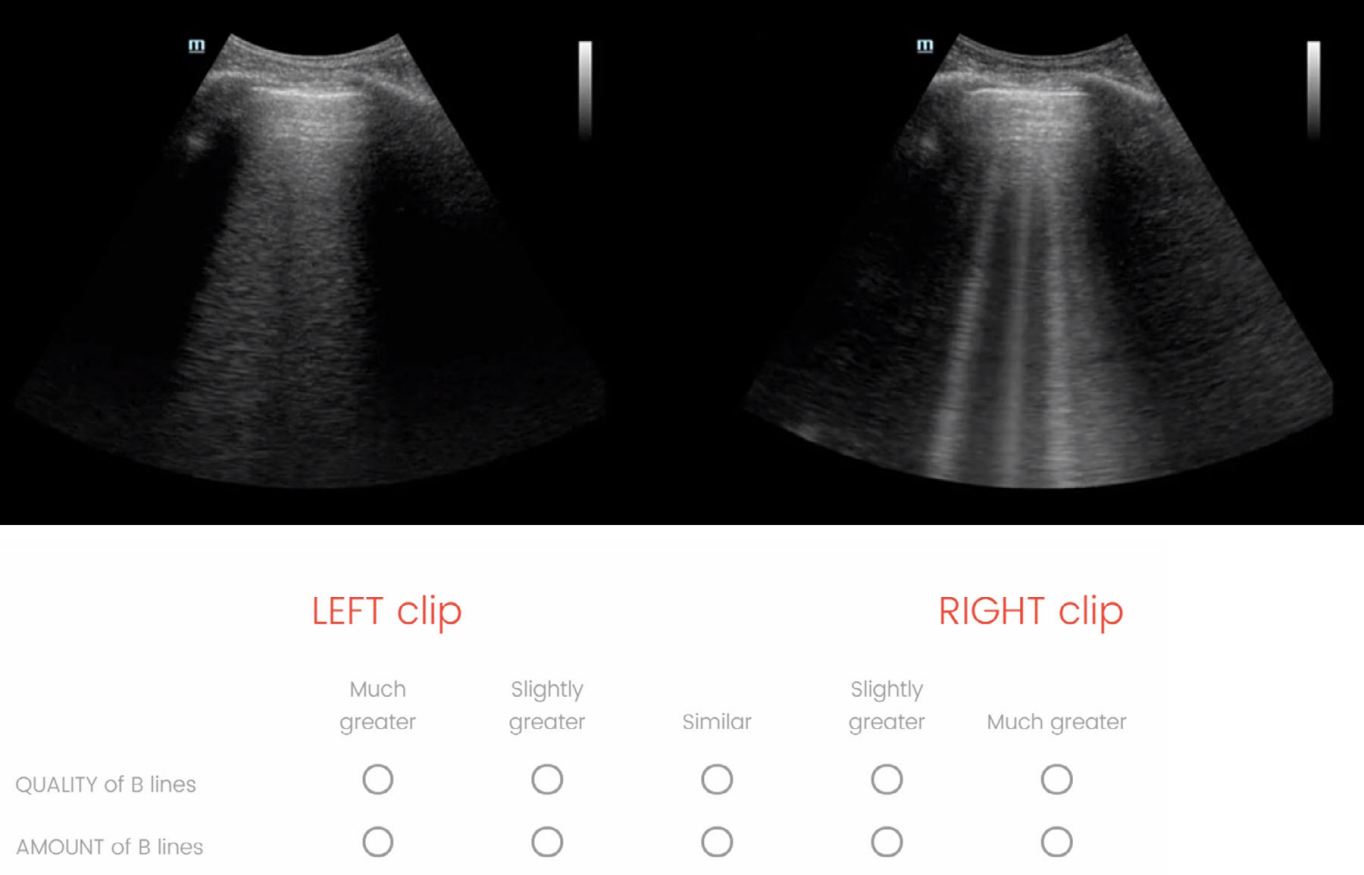
This is an image of the screen of one of the 40 clip‐pairs that were evaluated in the survey. Respondents were asked to rate the clips, left versus right, for quality and amount of B‐lines. These images were from a curvilinear probe, with the typical settings on the left and adjusted settings on the right. Video examples of the clips as seen by the respondents are available in the online supplement.

For each patient and probe type, the pair of clips obtained using Typical and Adjusted settings was juxtaposed on a single screen using video editing software (Camtasia, Techsmith, Okemos, MI) (see Figure 2). Coin flip randomization was used to determine which clip appeared on the left of the screen. All patient and machine settings data were removed. A convenience sample of 14 sonologists with experience in lung ultrasound (>100 exams) was recruited to evaluate the recorded clips. An online survey (Qualtrics, Provo, Utah) was created to record their assessments for each of the 40 pairs of clips. Respondents were asked to rate whether the right or left clip had “much greater,” “slightly greater,” or “similar” findings with respect to the quality and amount of B‐lines (Figure 2). Quality was described to the respondents as more easily distinguishable from background artifact and more clearly meeting the criteria for a B‐line. The sonologists came from specialty backgrounds in hospital medicine, critical care medicine, nephrology, and emergency medicine.

### Statistical analysis

Integer values from 1 to 5 were assigned to the B‐line ratings, with 1 assigned for typical settings “much greater,” 2 for typical settings “slightly greater,” 3 for both settings “similar,” 4 for adjusted settings “slightly greater,” and 5 for adjusted settings “much greater.” Mean values for the integer scale ratings were calculated. 95% confidence intervals for the mean values were calculated using a linear mixed effects regression model. Adjusted settings were determined to show significantly greater B‐line quality or quantity if the mean rating was greater than the null value of 3, and the 95% confidence interval did not cross 3. Statistical analyses were performed using R version 3.6.2.^26^

## Results

In our initial retrospective review 174 cardiopulmonary ultrasound exams were identified containing lung clips evaluating B‐lines. In these exams the focal zone was on average 6.1 centimeters deep to the pleura, and harmonics were on in 86.8% of exams. Details are given in Table 1, and sample clips of the typical and adjusted settings are available in the online supplement (Video 1, Video 2). The survey respondents who reviewed the images included 13 physicians and one ultrasound technologist with over 10 years of experience as a sonographer educator. Characteristics of the image reviewers are given in Table 2. Clinical characteristics of the 20 patients who were the sources of the prospectively collected pairs of clips are given in Table 3. Of the 20 patients, 16 had pulmonary edema, while 4 had interstitial lung disease.

**Table 1:** Retrospective review of cardiopulmonary ultrasound exams containing lung clips evaluating B‐lines

Total cardiopulmonary ultrasound exams with lung clips evaluating B‐lines from January 2019 to June 2019	174
Probe	
‐ curvilinear	52 (29.9%)
‐ phased array	122 (70.1%)
Preset	
‐ lung	63 (36.2%)
‐ cardiac	87 (50.0%)
‐ abdomen	20 (11.5%)
‐ focused assessment with sonography for trauma (FAST)	3 (1.7%)
Mean focal zone depth from pleura, centimeters, +/− standard deviation	6.1 +/− 3.1
Harmonics on	151 (86.8%)

**Table 2:** Details of the image reviewers

Total reviewers	14
Highest level of ultrasound training	
‐ Ultrasound technologist undergraduate degree	1
‐ Completed POCUS fellowship	5
‐ Current POCUS fellow	4
‐ Component of residency training	4
Clinical role	
‐ Sonographer educator	1
‐ Emergency medicine attending physician	9
‐ Pulmonology and critical care attending physician	1
‐ Pulmonology and critical care fellow physician	1
‐ Internal medicine hospitalist attending physician	2

**Table 3:** Clinical characteristics of patients who underwent prospective lung ultrasound

Total patients who underwent prospective lung ultrasound evaluating B‐lines	20
Female gender	9
Body mass index, median (interquartile range)	25.6 (21.8–27.7)
Chest wall thickness at right second intercostal space, centimeters, median (interquartile range)	1.67 (1.35–2.05)
Age, years, mean, +/− standard deviation	66.2 +/− 13.5
Diagnosis	
‐ Acute decompensated heart failure	10
‐ End stage renal disease	6
‐ Interstitial lung disease	4

Results of the B‐line ratings are given in Table 4. Adjusted settings B‐line quality was rated “slightly greater” or “much greater” more often than typical settings for both curvilinear (96.8% vs 0.4%) and phased array probes (78.2% vs 7.5%). Adjusted settings B‐line quantity was rated “slightly greater” or “much greater” more often than typical settings for both curvilinear (73.9% vs 2.1%) and phased array probes (56.1% vs 6.4%).

**Table 4:** Ratings of B‐line quality and quantity in 20 pairs of clips using a curvilinear probe and 20 pairs of clips obtained with a phased array probe

	*B* − *line Quality*	*B* − *line Quantity*		
	Curvilinear	Phased array	Curvilinear	Phased array
**Total ratings**	280	280	280	280
**Adjusted settings much greater**	200 (71.4%)	91 (32.5%)	126 (45%)	53 (18.9%)
**Adjusted settings slightly greater**	71 (25.4%)	128 (45.7%)	81 (28.9%)	104 (37.1%)
**Both settings similar**	8 (2.9%)	40 (14.3%)	67 (23.9%)	105 (37.5%)
**Typical settings slightly greater**	0	19 (6.8%)	5 (1.8%)	17 (6.1%)
**Typical settings much greater**	1 (0.4%)	2 (0.7%)	1 (0.4%)	1 (0.4%)

Results of statistical significance testing for B‐line ratings are given in Table 5. On a 1 to 5 integer scale, with 1 representing typical settings “much greater” and 5 representing adjusted settings were “much greater,” the mean rating was greater than the null value of 3, and the 95% confidence interval did not cross 3 for all categories. This indicates adjusted settings were significantly greater in B‐line quality and quantity for curvilinear and phased array probes.

**Table 5:** Ratings of quality and quantity of B‐lines in 20 pairs of clips using a curvilinear probe and 20 pairs of clips obtained with a phased array probe. For this scale, 1 means typical settings were “much greater,” and 5 means adjusted settings were “much greater”

	Mean rating	95% confidence interval
**B‐line quality, curvilinear probe**	4.68	4.50–4.85
**B‐line quality, phased array probe**	4.02	3.70–4.35
**B‐line quantity, curvilinear probe**	4.16	3.84–4.49
**B‐line quantity, phased array probe**	3.68	3.41–3.96

The mean rating was higher for the curvilinear than the phased array probe in both B‐line quality (4.68, 95% confidence interval 4.50–4.85, vs 4.02, 95% confidence interval 3.70–4.35) and quantity (4.16, 95% confidence interval 3.84–4.49, vs 3.68, 95% confidence interval 3.41–3.96), indicating a trend towards increased effect of adjusted settings in the curvilinear probe. This trend only reached significance for B‐line quality, as the 95% confidence intervals for curvilinear and phased array probes overlapped for B‐line quantity.

## Discussion

This study found that experienced sonologists considered LUS images using published optimized machine settings (based on in vitro studies) were markedly superior, in both the perceived quality and quantity of B‐lines, to those generated using typical settings. This finding was more pronounced with the curvilinear probe than the phased array probe. As such, we recommend that LUS for B‐line assessment be performed with the focal zone at the level of the pleura, harmonics off, and gain increased in the far field.

B‐line assessment is of great interest to a wide range of specialists whose clinical practice requires the recognition and management of many common disorders. When B‐line counts are using to guide patient therapy over time, our study demonstrates that changes in settings could influence B‐line scoring unrelated to clinical change.

Previous studies have compared curvilinear and phased array probes for B‐line inter‐rater reliability or concordance with findings on computed tomography scan of the chest, but there are no data directly comparing these probes for B‐line quality and amount.^8^, ^16^, ^17^, ^21^, ^22^, ^27^ Our study did not seek to correlate B‐line scores with pathologic or clinical findings; rather it sought to determine whether particular settings generated a perceptually superior image with respect to the number and quality of B‐lines. For the many clinicians who seek the information rapidly accessible via LUS, but who have less experience with ultrasound, it would be of benefit to have defined settings that are known to facilitate the examination. It is also possible that this methodology could be used by manufacturers to generate optimal presets for B‐line assessment on their machines.

This study did not attempt to compare the Adjusted settings between different transducer types. From this study, it would be erroneous to conclude that, because the increase in the quality and quantity of B‐lines was more marked with the curved array transducer when using the Adjusted setting, the curved array generated better images than the phased array transducer. It is possible that the baseline (Typical) settings were superior in the phased array probe, leading to less of an improvement with the Adjusted settings. We also did not assess how this process would be affected by the use of a linear array transducer, which is another form of transducer occasionally used for B‐line assessment.^28^ Future study of B‐line visualization in curvilinear, linear, and phased array probes is needed, as many practice environments do not have every probe type available, due to cost or the logistical limitations of handheld ultrasound devices.

Our study had several limitations. Since we tested only two machine settings, it is impossible to determine from our data whether focal zone, harmonics, or time gain compensation was the most important setting to change. Our study patients had a median body mass index of 25.6, reflecting a notably lower rate of obesity, defined as body mass index greater than 30, than our local and national average rates of obesity, which are greater than 30%.^29^, ^30^ We also obtained all clips in the right second intercostal space in the midclavicular line, with resulting median chest wall thickness of 1.67 centimeters. It is possible that our findings would have been different in other locations or in patients with thicker or thinner chest walls. Our study did not attempt to assess whether the sonologists' perceptions of an improved image actually would result in a more accurate test. It is possible that seeing B‐lines more clearly, and in greater number, might “overcount” B‐lines, and thereby undermine previously published B‐line scoring methods, which were developed without clearly defined machine settings. However, it seems more likely that images that reveal B‐lines more clearly and in greater number will make the test easier to interpret in the not uncommon situation in which the clinician is uncertain whether the finding is B‐lines, Z‐lines, areas of confluent B‐lines, or areas of normal lung. It is also notable that there was wide agreement among the group of experienced sonologists in our study as to which settings gave rise to better images.

Our prospectively collected images were all obtained using a single model of one manufacturer's ultrasound machine. The lung preset includes a gray map as well as a dynamic range compression curve that will differ among manufacturers. Transducer characteristics and post processing algorithms also vary widely. With the setting optimizations established in this study, further research into manufacturer, device, and probe effects on B line visibility is indicated.

The generalizability of the findings in this study is limited by our use of “typical” settings at our institution as a comparator. Further research is needed into the optimal settings and probe type to view B‐lines.

## Conclusions

Experienced sonologists perceived that video images of B‐lines acquired with in vitro evidence‐based settings were significantly superior with respect to the quality and quantity of B‐lines compared to those obtained with unadjusted settings. When performing a lung ultrasound exam for B‐lines, this study suggests that focal zone should be placed at the level of the pleura, harmonics turned off, and gain increased in the far field.

## Supporting information


**Video 1** A clip of the lungs of a patient with acute decompensated heart failure using the typical settings.

**Video 2** A clip of the lungs of the same patient using the adjusted settings.
